# Case of Complete Procidentia Uteri Successfully Treated by Operation

**Published:** 1879-09

**Authors:** Nelson H. Church

**Affiliations:** 300 S. Halsted Street; Chicago, Ill.


### ARTICLE VIII.

### *Case of Complete Procidentia Uteri Successfully Treated by Operation.*

The following case may serve to illustrate the power of the the gynæcologist in restoring to a new life, as it were (by a comparatively simple operation), one who had suffered for more than three years, and whose happiness had nearly vanished because of the apparent hopelessness of her condition.

Mrs. C., æt. 42, came under my notice Aug. 30th, 1878. She is rather an intelligent lady; married twenty-three years (one husband), and had given birth to two children.

In answer to a summons, I found her in great pain, and upon examination as to the cause, discovered a tumor as large as my fist external to the labia. It was soft and fluctuating. Upon attempting to reduce it, my fingers encountered a hard body within the pouch, which proved to be the uterus. Upon attempting to pass the catheter, with the meatus urinarius external to the vulva, the instrument met with resistance, the canal being doubled upon itself; a small quantity of urine was drawn, thus proving the existence of vagino-cystocele. A finger introduced into the rectum passed down into the prolapsed pouch, thus proving vagino-rectocele. When the parts were in a state of prolapse — for I found the case one of long standing — of course the bladder and rectum were unable to discharge their contents, hence, retention of urine and constipation of the bowels were almost constant complaints. Whenever the patient desired to empty the rectum or bladder, the tumor had to be reduced. After the birth of her second child — for a period of three and a half months — her knees were involuntarily apart, during which time there was evidently a prolapsed state of the uterus, rectum and bladder. In the normal state, the uterus, together with some convolutions of the small intestines, are mainly kept *in situ* by support from the vagina. When the bladder is distended, it is kept from retroverting by resistance from the vaginal walls, while the same cause operates to prevent the anterior wall of the rectum from falling forward. When, however, the vaginal walls are impaired by disease or otherwise — as from enlargement of the uterus, vaginitis, etc. — the resistance is no longer sufficient to render proper support to the adjacent parts, in consequence of which the vagina falls upon itself, and we have what is known as "prolapsus vagina." It is a mooted question whether prolapsus of the uterus occasions prolapsus of the vagina. Dr. Barnes, in his "Diseases of Women," late edition, is of opinion that "prolapsus of the vagina may exist independently," and further states, that "there is a preparation in St. George's Museum, which seems to show that vaginal rectocele may exist without prolapse of the uterus." Dr. Savage ("Female Pelvic Organs") says: "Prolapse of the vagina alone, or prolapse of the vaginal mucous membrane alone, are two affections which, anatomically considered, would seem impossible." Dr. Thomas, in his "Diseases of Women," states that: "Noel mentions a case of this kind where the prolapse reached down to the knees." Dr. Thomas further remarks: "It is an important question whether there can be prolapse of the vagina without vagino-rectocele, vagino-cystocele, vaginal hernia of intestine forcing down the vaginal *cul-de-sac* or uterine prolapse." In the case of Mrs. C., I think all of the conditions mentioned by Dr. Thomas existed. Through a correspondence with a physician of Iowa, who formerly had charge of her case, I learned that her prolapsed state was greatly aggravated, if not entirely induced, by the insatiable lust of her husband, whose bestial approaches, when heightened by liquor, were as frequent as four or five times in a single night. This gentleman operated upon her twice, each operation proving a failure owing to the venereal appetite of her husband, who, Satyr-like, in the language of the doctor, "tore things all loose." From the very pointed language that the doctor employs in describing Mrs. C.'s case, one would naturally suppose that the sole trouble originated in the unnatural size of her husband's penis, and his amorous propensity for using it. Frequently it was necessary to inject morphine subcutaneously for relief of pain occasioned by excessive coitus.

The uterus seemed to be about twice its normal size, and within it there appeared to be an apparently hard, resisting body, while every five or six weeks there was a free discharge of muco-purulent matter, from which conditions I inferred there might possibly exist a fibroid. The error of this belief was made apparent at the time of an operation subsequently performed by Dr. Jackson, when upon careful examination under ether, no such growth was found.

The woman having separated from her husband, applied for admission, and was received into the Woman's Hospital, State of Illinois, Dr. A. Reeves Jackson in charge. In January, 1879, Dr. Jackson operated for narrowing the vagina, at which I was present. The patient was anæsthetized, placed in the lithotomy position, and the operation conducted under the Lister spray. The operator commenced by picking up a fold of the posterior and upper vaginal wall above the os uteri, and with a pair of scissors cutting out a circular portion 3.7 Cm. in diameter, introducing a running suture around the margin of the cut surface — a sort of puckering string — drawing the suture together at the ends and tying. The operation was skilfully and expeditiously performed.

The patient made good recovery, but the cure being incomplete, Dr. Jackson performed a second operation a few weeks later — the last operation being similar to the first, save that the lower and posterior portion of the vagina was acted upon.

The woman called upon me not long since; I examined her and found that the operation had completely cured her.

NELSON H. CHURCH, M.D.,

CHICAGO, ILL. 300 S. Halsted Street.

